# Shaping Long-term Care Insurance Intentions Among Chinese Adults Aged 50–70: Role of Information Interventions in Health Risks

**DOI:** 10.1093/geroni/igaf054

**Published:** 2025-05-24

**Authors:** Jin Liu, Jiaozhi Hao, Elizabeth Maitland, Stephen Nicholas, Jian Wang, Anli Leng

**Affiliations:** School of Political Science and Public Administration, Shandong University, Qingdao, China; School of Political Science and Public Administration, Shandong University, Qingdao, China; School of Management, University of Liverpool, Liverpool, UK; Australian National Institute of Management and Commerce, Sydney, NSW, Australia; Newcastle Business School, University of Newcastle, Newcastle, NSW, Australia; Dong Fureng Institute of Economic and Social Development, Wuhan University, Wuhan, China; Center for Health Economics and Management at School of Economics and Management, Wuhan University, Wuhan, China; School of Political Science and Public Administration, Shandong University, Qingdao, China; Smart State Governance Lab, Shandong University, Qingdao, China

**Keywords:** Dementia, Disability, Health risk communication

## Abstract

**Background and Objectives:**

China’s population aging, especially increasing numbers of older people with disability and dementia, challenges the public health care system. Long-term care insurance (LTCI) is essential to provide care for China’s fast-growing aging population, yet there is a lack of evidence on how the health risks of disability and dementia influence the acceptance of LTCI in China. This study explores the effects of health risk information about disability or dementia on the LTCI intentions of the Chinese aged 50–70.

**Research Design and Methods:**

Using stratified random sampling and convenience sampling methods, we designed a survey of 1 025 respondents aged 50–70 from 8 provinces in China. We randomly assigned respondents to control (*n* = 354), disability risk (*n* = 339), and dementia risk (*n* = 332) groups, and used multinomial logit models to investigate the information intervention effects on respondents’ different LTCI (No/Uncertain/Yes) intentions. Also, we analyzed the heterogeneity of respondents’ education level and whether living in LTCI pilot/nonpilot policy cities.

**Results:**

Both disability and dementia risk information significantly decreased the probability that respondents were unwilling relative to being willing to enroll in LTCI. The marginal prediction results show that pretest LTCI intentions played a key role in shaping postintention LTCI. In the analysis of heterogeneity, we found that disability information was associated with an increased likelihood of respondents with lower levels of education answering “uncertain,” and dementia information was associated with a decreased likelihood of answering “no” for respondents living in the LTCI pilot policy cities.

**Discussion and Implications:**

Disability and dementia risk interventions significantly improved LTCI intentions among Chinese aged 50–70. To improve the acceptance of LTCI, we recommend that policymakers adopt the strategies of information interventions for LTCI policy advocacy and differentiate dementia-related and disability-related risk information by individuals with different educational levels and residents in LTCI pilot/nonpilot policy cities.

**Translational Significance:** We explored the relationship between the health risk information intervention in disability and dementia and the change in long-term care insurance (LTCI) intentions among Chinese adults aged 50–70. We found that disability and dementia risk information had positive effects on improving respondents’ LTCI intentions, and the effects differed according to individuals’ educational levels and living in LTCI pilot cities. These findings provide policymakers with feasible and promising strategies for increasing the acceptance and coverage of LTCI, which will help to achieve person-centered health care and improve the quality of life of Chinese residents.

## Background and Objectives

With the world’s fastest aging population, China’s increasing life expectancy and declining birth rates challenge the provision of healthcare (1). In 2020, there were 264 million people aged 60 years old and over, accounting for 18.7% of the total population, forecast to rise to more than 500 million by 2050 (2,3). The number of years that older people live with disability is also continuing to grow, from 5.78 years in 2015 to projected 7.44 years in 2030 and is expected to reach 11.45 years in 2050 (4). The number of over 60-year-old Chinese with dementia was 15.07 million in 2018 and is expected to rise to 50 million by 2050 (5,6). Older adults with disabilities and dementia pose a serious challenge to the sustainability of China’s long-term care (LTC) system (7,8). With the reduction in the size of the Chinese family, the effects of the 1-child policy, urban migration, and the decline in informal family-provided LTC, the demand for formal LTC has surged (9).

To address this challenge, the Chinese government launched a long-term care insurance (LTCI) scheme in 2016, called the sixth social insurance, in 15 pilot cities, expanded to 49 cities by 2020, to provide accessible and affordable LTC services for old people with severe disabilities, measured by their ability to perform activities of daily living (10). Long-term care insurance aimed to alleviate the shortage of caregivers for severely disabled patients by providing privately funded nursing service payments in public care facilities and some support for home-based care services (11). Covering 183 million people in 2023, the LTCI program provided an average of RMB8800 (US$1 250) in reimbursements per beneficiary, representing 22.5% of China’s average per capita disposable income (12). With minor differences between pilot cities, LTCI was open to employees and retirees enrolled in Urban Employee Basic Medical Insurance (UEBMI), and for a few cities, urban and rural residents enrolled in Urban and Rural Residents Basic Medical Insurance (URRBMI). Currently, China’s National Healthcare Security Administration is establishing a nationwide and unified LTC social insurance system independent from medical health insurance (13). As key participants in the current and future LTCI programs, it is critical to understand preferences for, and to increase the uptake of, LTCI among older Chinese residents.

We analyze LTCI purchasing decisions from the economic, behavioral, and psychosocial perspectives (14–18). Behavioral economics provides a powerful framework for understanding the purchase decision behavior for LTCI by focusing on individual risk perceptions in intertemporal health decision-making choices (19). An individual’s risk perception, derived from objective knowledge of LTC risks, subjective knowledge of LTC risks, and subjective knowledge of one’s own health, has been shown to shape LTCI needs (20–22). Cognitive biases or overconfidence mean individuals’ health cognition may deviate from rational expectations, resulting in a misunderstanding of LTCI risks affecting decision-making (23,24). Individual LTCI perceptions biases reflect physical, mental, financial, and other factors associated with the future projection of disability or dementia in old age (25,26). When confronted with new information, information processing theory holds that individuals encode, store, and extract external information and make decisions based on the information (27). Motivated by self-protection, the probability of individuals purchasing LTCI increases with their knowledge of the risks associated with their future likely LTC needs (28). These studies suggest that expectations of LTC risk are positively correlated with changes in LTCI demand, and it is possible to promote individuals’ participation in LTCI by increasing their risk perception.

In healthcare research, these behavioral hypotheses have been widely applied to insurance enrollment, vaccination decisions, and medication use to facilitate the public’s health-favorable decision-making (29–31). Surprisingly, there is currently a lack of research on the role of information interventions in terms of disability and dementia health risks in shaping the LTCI intentions of Chinese aged 50–70. To address this research gap, we designed a survey experiment to explore whether Chinese adults aged 50–70 with information about the adverse health outcomes of disability or dementia would influence their intentions to LTCI insure. We hypothesize that disability and dementia information significantly shape individuals’ LTCI intentions. Since our study is concerned with the effect of information interventions on respondents’ LTCI intentions, the results are expected to vary depending on the individual’s own risk perceptions. Participants’ education background and whether they reside in an LTCI pilot city proxy respondents’ knowledge about LTCI (32–34), so our study conducted a heterogeneity analysis to explore the intervention effects of disability and dementia information for subgroups with different education levels and living in LTCI pilot/nonpilot policy cities.

Our study makes contributions in the following 3 aspects. From the perspective of risk perception, we explore whether increasing people’s understanding of the risk of disability or dementia could influence their intentions to participate in LTCI. This not only enriches the research on information intervention in health promotion but also provides empirical references for the Chinese health policymakers to improve the LTCI coverage rate of residents. Second, we examine the heterogeneity of LTCI knowledge on LTCI decision-making, providing evidence for the implementation of individualized and effective interventions. Finally, our study provides health policymakers with evidence on LTCI intentions in China, helping improve China’s “person-centered” LTC system to tailor LTCI needs to the preferences of individuals.

## Research Design and Methods

### Data and Sample

To examine the relationship between disability and dementia information interventions and the change of LTCI intentions among adults aged 50–70 in China, we conducted a random survey experiment in August 2022. Using the stratified random sampling and convenience sampling methods, we randomly selected 8 provinces that were broadly representative of eastern (Shandong, Jiangsu, Zhejiang), central (Anhui, Henan, Hubei), and western (Shaanxi, Yunnan) regions of China. Between 1 and 3 LTCI pilot and nonpilot cities were randomly selected in each province, and 100 individuals were randomly recruited and interviewed face-to-face by trained interviewers using convenience sampling in each city.

The inclusion criteria were respondents aged between 50 and 70, without cognitive impairments. There are 2 main reasons why we selected adults aged 50–70 for our survey. First, 50–70 years old is the transition period into old age, and this age group is in a critical period of gradually increasing LTC needs. Conducting a survey on adults aged 50–70 to understand their LTC intentions will help to provide evidence for future LTCI policy formulation and service planning. Second, adults aged 50–70 typically have greater cognitive and decision-making abilities and are more likely to understand and respond to survey questions than those at a more advanced age.

A total of 1 172 questionnaires were distributed, of which 1 025 respondents answered all the options, with a response rate of 87.46%. Respondents were informed about the study, allowed to withdraw at any stage, and gave informed consent.

### Procedure

Our experiment was randomized by setting up different questionnaire scenarios. Three versions of the anonymous questionnaire were created on the online questionnaire website credamo (credamo.com) by a researcher who was not the investigator, and a random assignment procedure was built into the questionnaires, which can help investigators randomly assign participants to one of the 3 groups (control group, disability treatment group, and dementia treatment group). There were 4 parts contained in the survey questionnaire: Part 1 collected basic personal information, including their basic information (sex, age, marital status, urban–rural residence, education level, and number of children), self-reported health status, mental health, and physical health; Part 2 measured pretesting of LTCI intentions; Part 3 provided a brief introduction about the LTCI scheme for all participants and additional information for the 2 treatment groups; and Part 4 retesting of LTCI intentions in the control group with the identical question as in Part 2 and in the 2 treatment groups by asking participants to put themselves in the shoes of the older adults in the disability or dementia scenario in Part 3. To highlight the important information in both disability and dementia scenarios to the surveyed older adults, we underlined the important descriptions in the questionnaire. Before answering Part 4, the control and treatment groups were provided with the same information about the LTCI scheme:

*“Long-term care insurance (known as the ‘sixth insurance’ of social insurance) is an institutional arrangement that focuses on providing care guarantee and financial compensation when the insured person loses the daily living ability, falls ill or dies in old age. In 2016, long-term care insurance began to be piloted in China, targeting insured people in long-term incapacity and focusing on solving the costs required for basic living care and medical care for people with severe incapacity.”*

In addition, the disability treatment group (but not the control group) read the following disability scenario before answering Part 4:

*“An 80-year-old person was able to eat, wash, go to the toilet or get dressed on his/her own. However, his/her bladder and bowel functions were weakening and he/she gradually became incontinent. After a broken bone, he/she has difficulty walking and currently requires a wheelchair, and is unable to bathe and go outdoors independently. Family members are unable to provide adequate care support for the older person due to their own health problems or work needs.”*

And the dementia treatment group (but not the control group) read the following dementia scenario before answering Part 4:

*“An 80-year-old person is able to eat, wash, go to the bathroom, dress or walk on his/her own. However, his/her bladder and bowel functions were weakening and he/she gradually became incontinent. With Alzheimer’s disease (which is a form of dementia), there are some symptoms of dementia, such as significant memory loss, forgetting whether or not they have eaten or taken their medication, forgetting to turn off the gas, and it is becoming increasingly difficult to control with drugs, and they are unable to bathe independently and go outdoors to be active. Family members are unable to provide adequate care support for the older person due to their own health problems or work needs.”*

### Measures

The dependent variable was pretest LTCI intention, assessed through the question “Would you like to enroll in long-term care insurance?,” which was measured by the question “No” (0), “Uncertain” (1), and “Yes” (2) response options.

As shown in Table 1, the control variables comprised individual sociodemographic characteristics, measured by sex (male/female), age (50–59/60–69), marital status (no spouse/have a spouse), urban–rural residence, monthly income (less than 3 000 yuan/3 000–6 000 yuan/more than 6 000 yuan), education level (less than 6 years/6–9 years/more than 9 years), LTCI pilot/nonpilot city, UEBMI (enroll in UEBMI/don’t enroll in UEBMI), number of children (1/2 and more), self-rated health status (good/not good), physical health, and mental health. Physical health and mental health were measured by the activity of daily living (ADL/IADL) scale and ICECAP-O scale, as shown in Supplementary Tables 1 and 2 in Supplementary Material. The numbers 1–4 represented the scores for each item, and we added up the scores of each item for each respondent. To eliminate the impact of dimensions, we standardized the scores to derive the final physical health and mental health scores of each respondent.

**Table 1.** Characteristic of Respondents

Variables	Total (*n* = 1 025)		Control Group (*n* = 354)		Disabled Group (*n* = 339)		Dementia Group (*n* = 332)		*p*
Sex, *n* (%)									
Female	559	(54.54)	214	(60.45)	167	(49.26)	178	(53.61)	.012
Male	466	(45.46)	140	(39.55)	172	(50.74)	154	(46.39)	
Age, *n* (%)									
50–59	572	(55.80)	161	(45.48)	221	(65.19)	190	(57.23)	<.001
60–69	453	(44.20)	193	(54.52)	118	(34.81)	142	(42.77)	
Marital status, *n* (%)									
No spouse	146	(14.24)	52	(14.69)	46	(13.57)	48	(14.46)	.907
Have a spouse	879	(85.76)	302	(85.31)	293	(86.43)	284	(85.54)	
Residence, *n* (%)									
Rural	383	(37.37)	138	(38.98)	129	(38.05)	116	(34.94)	.523
Urban	642	(62.63)	216	(61.02)	210	(61.95)	216	(65.06)	
Educational level, *n* (%)									
Low (≤ 6 years)	300	(29.27)	167	(47.18)	51	(15.04)	82	(24.70)	<.001
Medium (6 to ≤ 9 years)	269	(26.24)	92	(25.99)	93	(27.43)	84	(25.30)	
High (> 9 years)	456	(44.49)	95	(26.84)	195	(57.52)	166	(50.00)	
Living in a LTCI pilot city, *n* (%)									
Yes	439	(42.83)	127	(35.88)	113	(33.33)	199	(59.94)	<.001
No	586	(57.17)	227	(64.12)	226	(66.67)	133	(40.06)	
UEBMI, *n* (%)									
Yes	348	(33.95)	96	(27.12)	119	(35.10)	133	(40.06)	.001
No	677	(66.05)	258	(72.88)	220	(64.90)	199	(59.94)	
Monthly personal income (RMB), *n* (%)									
1 (≤ 3 000)	404	(39.41)	194	(54.80)	73	(21.53)	137	(41.27)	<.001
2 (3 000–6 000)	379	(36.98)	126	(35.59)	133	(39.23)	120	(36.14)	
3 (> 6 000)	242	(23.61)	34	(9.60)	133	(39.23)	75	(22.59)	
Number of children, *n* (%)									
1	405	(39.51)	92	(25.99)	131	(38.64)	182	(54.82)	<.001
>=2	620	(60.49)	262	(74.01)	208	(61.36)	150	(45.18)	
Self-rated health status, *n* (%)									
Not good	336	(32.78)	123	(34.75)	94	(27.73)	119	(35.84)	.051
Good	689	(67.22)	231	(65.25)	245	(72.27)	213	(64.16)	
Physical health (mean, *SD*)	0.941	(0.131)	0.933	(0.102)	0.918	(0.182)	0.973	(0.081)	<.001
Mental health (mean, *SD*)	0.672	(0.194)	0.603	(0.176)	0.679	(0.182)	0.738	(0.199)	<.001

*Notes*: LTCI = long-term care insurance; RBM = renminbi (Chinese Yuan); UEBMI = Urban Employee Basic Medical Insurance.

### Analytic Strategy

All data processing and analyses were performed with the use of STATA 16.0. First, we used ANOVA and Pearson chi-square tests to compare 3 groups (control group, disability treatment group, and dementia treatment group) on sociodemographic characteristics and the pretest and post-test LTCI intentions among the 3 groups. Second, to examine the effects of information interventions on post-test LTCI intention, multinomial logistic regression models were constructed. To mitigate confounding by initial differences of respondents, pretest LTCI intention was added to the models as a control variable. The marginal effects of the information interventions were also analyzed according to different baseline pretest LTCI intentions. Then, we conducted heterogeneity analysis among respondents with different educational levels and living in LTCI pilot/nonpilot cities to compare the intervention effects across subgroups. All models included control variables, and the results were reported with 95% confidence intervals using relative risk ratio (RRR; which indicates the multiple of the relative risk change in a category of the dependent variable relative to the base category) and predictive margins (which indicates the impact of changes in an independent variable on the probability of each category of the dependent variable).

## Results

### Respondent Characteristic

Table 1 shows the respondents’ socioeconomic demographic characteristics. Out of the 1 025 respondents, 466 (45.46%) were male, 572 (55.80%) were between the ages of 50 and 59, and 456 (44.49%) had a high education level. Most had a spouse (85.76%), were urban residents (62.63%); did not live in an LTCI pilot policy city (57.17%) and did not have UEBMI (66.05%); earned less than RMB6000 a month (76.39%); and had 2 or more children (60.49%). In terms of the health status, 689 (67.22%) respondents rated their own health status as good. After 0–1 standardization, the average physical health status was a high 0.94 level, and the mental health status was a moderate 0.67 level. The last column of Table 1 shows the ANOVA results. There were significant intergroup differences in age, educational level, monthly personal income, LTCI pilot city, UEBMI, number of children, physical health, and mental health. To control the influence of these variables on the intervention results, they were added to the statistical model as control variables.

### Respondents’ LTCI Intentions

**Figure 1.**
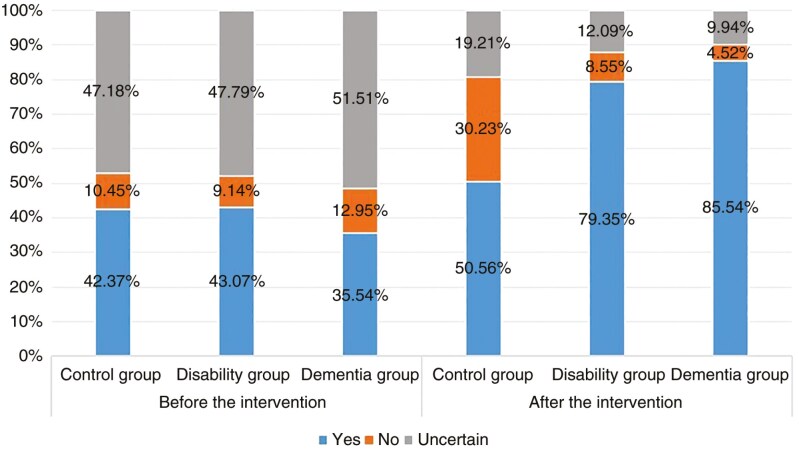
Comparison of LTCI intentions rates before and after the information intervention. *Note*: LTCI = long-term care insurance.

Figure 1 presents the respondents’ percentages of LTCI intentions in the control, disability, and dementia groups before and after the scenario intervention. Before the information intervention, the proportion of respondents who were willing, unwilling, and uncertain about intentions to LTCI was roughly a 4:1:5 ratio, which changed to a 7:2:1 ratio after the information intervention. The percentage of respondents who were willing to insure LTCI increased substantially in the disability group (43.07–79.35%) and dementia group (35.54–85.54%).

We also analyzed the LTCI intention before and after the scenario interventions by ANOVA analysis and used Bartlett’s test to examine the equal variance. The results show that before the information interventions, there was no significant intergroup difference in LTCI intentions in the control, disability, and dementia groups (*F* = 1.59, *p* = .204). However, the differences became statistically significant after the interventions (*F* = 39.45, *p* < .001), indicating that the scenario interventions affected LTCI intentions.

Table 2 shows the matrix of LTCI intention transfers before and after the information intervention among the 3 groups. The diagonal corresponds to respondents whose LTCI intentions remain unchanged. In the control group, most respondents’ LTCI intentions were unchanged or changed from “Uncertain” to “No.” Following the intervention, a majority of participants in both the disability and dementia groups changed their willingness to enroll in LTCI, surpassing the proportion of those who responded with “uncertain” or “no.” Results of the Pearson chi-square tests illustrate that there were significant differences between pretest and post-test LTCI in the 3 groups. The significant difference in the control group indicates that it is necessary to analyze the intervention effects with the control group as the reference.

**Table 2.** Matrix of LTCI Intention Transfers Before and After the Intervention

Group	Before the Intervention	After the Intervention			Chi2
		Yes	Uncertain	No	
Control group	Yes	139 (92.67%)	5 (4.00%)	6 (3.33%)	196.075^‡^
	Uncertain	5 (20.96%)	26 (33.53%)	6 (45.51%)	
	No	35 (13.51%)	76 (16.22%)	56 (70.27%)	
Disability group	Yes	124 (84.93%)	11 (7.53%)	11 (7.53%)	10.275^†^
	Uncertain	24 (74.69%)	5 (17.28%)	2 (8.02%)	
	No	121 (77.42%)	13 (6.45%)	28 (16.13%)	
Dementia group	Yes	111 (94.07%)	3 (3.39%)	4 (2.54%)	17.510^‡^
	Uncertain	30 (83.63%)	5 (12.28%)	8 (4.09%)	
	No	143 (69.77%)	7 (18.60%)	21 (11.63%)	

*Notes*: LTCI = long-term care insurance.

^*^Significant at 1% level.

^†^Significant at 5% level.

^‡^Significant at 10% level.

### Main Results of Multinomial Logistic Regression Models

Controlling for the pretest intentions, Table 3 shows the RRRs of disability and dementia information on the post-test LTCI intentions. The results indicate that pretest LTCI intentions had a significant effect on the transfer of the LTCI intention, and respondents whose pretest intentions were “yes” and “uncertain” had a lower relative risk of changing their intentions compared to respondents who answered “no.” For the disability intervention group, the relative risk of answering “no” decreased by a factor of 0.090 (CI: 0.048, 0.169, *p* < .01) compared to answering “uncertain” and decreased by a factor of 0.262 (CI: 0.130, 0.530, *p* < .01) compared to answering “yes.” For the dementia group, the relative risk of answering “no” decreased by a factor of 0.054 (CI: 0.027, 0.104, *p* < 0.01) compared to answering “uncertain” and decreased by a factor of 0.310 (CI: 0.146, 0.662, *p* < 0.01) compared to answering “yes.” In addition, both in the disability and dementia group, the relative risk of answering “uncertain” increased by a factor of 2.905 (CI: 1.720, 4.904, *p* < .01) and 5.799 compared to answering “yes” (CI: 3.356, 10.019, *p* < .01), respectively.

**Table 3.** Effects of Information Interventions on Post-test LTCI Intention: Multinomial Logit Regression Results

Variables	(1) Uncertain versus (2) Yes	(0) No versus (2) Yes	(0) No versus (1) Uncertain
	RRR	RRR	RRR
Disability intervention	2.905^‡^	0.262^‡^	0.090^‡^
	(1.720–4.904)	(0.130–0.530)	(0.048–0.169)
Dementia intervention	5.799^‡^	0.310^‡^	0.054^‡^
	(3.356–10.019)	(0.146–0.662)	(0.027–0.104)
Preintention (reference: no)			
Yes	6.369^‡^	0.234^‡^	0.037^‡^
	(2.960–13.703)	(0.092–0.597)	(0.017–0.078)
Uncertain	0.821	0.356^‡^	0.434^‡^
	(0.432–1.560)	(0.176–0.719)	(0.242–0.777)
Sex	1.160	1.225	1.056
	(0.772–1.742)	(0.734–2.044)	(0.679–1.642)
Age	0.690*	0.754	1.092
	(0.452–1.054)	(0.447–1.272)	(0.697–1.712)
Marriage status	0.586	0.566	0.965
	(0.308–1.115)	(0.269–1.187)	(0.539–1.729)
Educational level (reference: low)			
Medium	0.767	0.935	1.219
	(0.444–1.324)	(0.481–1.815)	(0.682–2.179)
High	0.712	1.262	1.773*
	(0.386–1.311)	(0.597–2.665)	(0.924–3.403)
LTCI polite policy	0.613^†^	0.464^†^	0.757
	(0.388–0.968)	(0.251–0.858)	(0.442–1.295)
UEBMI	1.631*	1.198	0.735
	(0.977–2.722)	(0.612–2.345)	(0.409–1.320)
Urban–rural residence	0.748	0.588*	0.786
	(0.464–1.204)	(0.326–1.060)	(0.471–1.312)
Monthly personal income (reference: ≤ RMB 3 000)			
2 (RMB 3 000–6 000)	1.474	1.258	0.853
	(0.889–2.443)	(0.678–2.333)	(0.497–1.465)
3 (≥RMB 6 000)	1.339	1.058	0.790
	(0.683–2.627)	(0.414–2.705)	(0.347–1.796)
Number of children	1.821^†^	1.356	0.745
	(1.139–2.910)	(0.738–2.490)	(0.433–1.280)
Self-rated health status	1.754^†^	1.259	0.718
	(1.126–2.731)	(0.729–2.174)	(0.444–1.160)
Physical health	0.881	0.038^‡^	0.043^‡^
	(0.121–6.421)	(0.004–0.325)	(0.010–0.184)
Mental health	2.285	1.209	0.529
	(0.683–7.642)	(0.265–5.508)	(0.140–1.995)
Constant	0.969	151.938^‡^	156.725^‡^
	(0.125–7.548)	(16.006–1,442.279)	(29.424–834.784)
Pseudo *R*^2^	0.2378		
Observations	1 025		

*Notes*: LTCI = long-term care insurance; RBM = renminbi (Chinese Yuan); RRR = relative risk ratio; UEBMI = Urban Employee Basic Medical Insurance. Confidence intervals in parentheses.

^*^Significant at 1% level.

^†^Significant at 5% level.

^‡^Significant at 10% level.

To compare the differences in the effects of disability and dementia information, we conducted a series of Wald tests. The results show that there was a significant difference only under the model of “(1) uncertain versus (2) yes” (chi2 = 5.84, *p* = .016), which indicates that information provided by disability and dementia groups differed in the extent to which respondents answered “uncertain” compared to “yes.” Further, we examined the relationship between the difference (RRR of disability information subtracts the RRR of dementia information) and 0 using the Lincom test. The results show that the difference is significantly greater than 0 (*p* = .016), suggesting that disability information, compared to dementia information, increases the probability of the respondents answering “uncertain” relative to “yes” by a larger amount.

**Figure 2.**
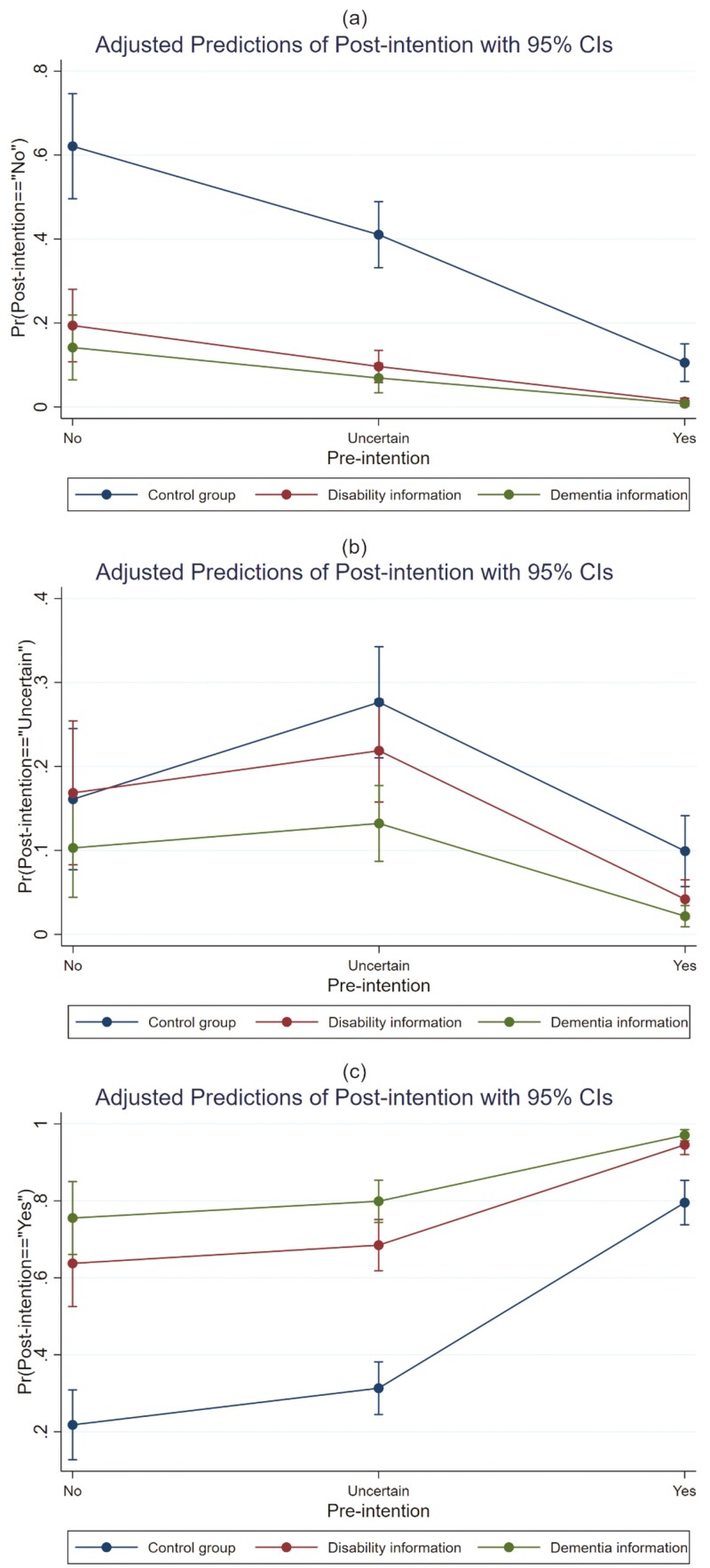
Adjusted predictions of “No” (A), “Uncertain” (B), and “Yes” (C) postintention for different pretest intentions and intervention groups.

As shown in Figure 2, we estimated the marginal effects of the multinomial logistic regression models’ adjusted prediction of postintention answering “no” (a), “uncertain” (b), and “yes” (c) under different combinations of pretest LTCI intention and intervention groups. Figure 2A shows that the adjusted prediction of postintention answering “no” presents a downward trend when pretest intentions change from “no” to “uncertain” to “yes,” and the marginal effects of disability and dementia are lower than those of the control group. For the prediction of post-test intention answering “uncertain” in Figure 2B, it is still the control group that has the largest marginal effect, and the marginal effect for each group is maximized when the pretest intention is “uncertain.” Figure 2C shows that the adjusted prediction of post-test intention answering “yes” increases as the pretest LTCI intention changes from “no” to “yes” and has the greatest marginal effects in the dementia information group, followed by the disability information group and the control group.

### Heterogeneity Analysis

Supplementary Table 3 presents the intervention effects and confidence intervals of disability and dementia information with different educational levels, and Supplementary Table 4 for different LTCI pilot cities. The results showed that the disability information had significant effects on the postintention of “no” for all educational levels, while the effects on the postintention of “uncertain” were only significant in the lowest educational level. For dementia information, the effects were statistically significant on the postintention of “uncertain” for all levels of education, but not significant on the answer of “no” in the medium and high educational level groups. The results indicated that lower educational levels were associated with a higher possibility that respondents answered “uncertain”, especially for the dementia information. Supplementary Table 4 reveals that disability information had significant intervention effects for all models within LTCI pilot cities and non-LTCI pilot cities, and dementia information was also significant for all surveyed cities except in the model that predicted “no” post-test LTCI intention in the non-LTCI pilot cities.

## Discussion and Implications

Conducting a randomized survey experiment, we analyzed the effect of different types of information intervention on shaping LTCI intentions among 50- to 70-year-old Chinese adults. After the scenario intervention, more than a third of respondents changed their LTCI intentions from unwilling or uncertain to willing, including 40.50% in the disability group and 48.32% in the dementia group. Only 11.17% in the control group shifted to a willingness to LTCI insure. After the information intervention, 71.41% of respondents were willing to LTCI insure, much higher than 40.39% before the experiment. These percentages are consistent with an intervention survey conducted in 2 pilot LTCI Chinese cities (35) and previous international studies on LTCI, health insurance, and catastrophic insurance that found information interventions increased people’s intentions to enroll in insurance (23,28,36). In addition, we created matrix plots to observe the transfer of LTCI intentions before and after information interventions for each group, which showed a significant increase in the proportion of respondents who chose “yes” after the intervention for the 3 groups of respondents. These descriptive results show that with awareness-raising measures, it is possible for the government policymakers to increase the acceptance and support of LTCI policy among middle and old-age adults in China.

In the context of sample characteristics, the respondents in our survey were between the ages of 50 and 70 with no cognitive impairment. It has been shown that there is an association between midlife behaviors and a range of later life outcomes, such as disability, dementia, and frailty (37), which increase the likelihood of requiring LTCI needs in the near future. However, many people may not be fully aware of the potential risks and subsequent financial and caregiving burdens they might face in their old age, underestimating the cost of LTC (38). The main multinomial logistic regression results show that both disability and dementia risk information significantly decreased the probability of the postintention of “no,” after controlling for the pretest LTCI intentions and other relevant socio-demographic characteristics. Moreover, by estimating the effect of the information intervention on post-test intentions across different pretest responses, we found that the marginal effect of the dementia and disability information on post-test responses of “yes” was much higher than that of the control group. These findings are in line with previous studies demonstrating that risk communication strategies in public health policy implementation can effectively modify individuals’ risk perception and subsequent behavioral intentions through the dissemination of evidence-based risk information (39,40). Through providing detailed information about disability and dementia risks in old age, policymakers and relevant institutions can help individuals better appreciate the importance and necessity of LTCI and increase the acceptance and uptake of LTCI, ultimately improving the well-being of older adults and the stability of the social security system.

We found that compared with the dementia information, the disability intervention would increase the probability of the respondents answering “uncertain” relative to “yes” by a larger amount, indicating that the dementia information is more effective in promoting people’s uptake of LTCI. These differential responses reflect disparity in risk assessments across types of health risk information, consistent with behavioral economics frameworks emphasizing the role of risk perception in intertemporal health-related decision-making (19). Specifically, the disparity might be due to more stigma being attached to dementia than disabilities, and that people show anxiety about loss of self-identity and dignity related to dementia (41). Also, the experiment material for the dementia scenario contained the physical incapacities that are necessary to qualify as an LTCI beneficiary, perhaps contributing to a higher coefficient of the dementia group than the disability group. Based on the difference between the effects of disability and dementia information, we recommend that policymakers and LTCI promoters consider these nuances when designing targeted communication strategies. It is essential to provide clear and detailed information about health risks and practical examples of how LTCI can support individuals and their families in dealing with disability and dementia challenges to help them make the best informed decisions.

We also examined the effect of information interventions on individuals with different levels of education and living in different LTCI pilot/nonpilot cities. The results show that lower educational levels were associated with a higher probability that respondents answered “uncertain,” especially for the dementia information group. Information processing theory argues that different levels of education are related to greater knowledge and information-processing capacities when processing new information (42). As suggested by information processing theory, individuals with higher levels of education had higher health literacy (43) and were more willing to enroll in insurance schemes to mitigate the health risks (40), enabling them to better evaluate and respond to health risk information. Subgroup regressions for different LTCI pilot cities indicate that dementia information was associated with a decreased likelihood of answering “no” for respondents living in the LTCI pilot policy cities. This finding again aligns with information processing theory, which highlights contextual factors, such as exposure to information, in shaping people’s responses to decision-making (44). In LTCI pilot cities, where the government promotes, interprets, and practices LTCI policies through the media or in the community, people were more likely to gain more knowledge related to aging and health than residents of nonpilot cities, which will influence pilot city residents’ LTCI decisions (45). In our survey results, 19.82% of respondents living in the pilot cities indicated that they were aware of the LTCI policy, which is significantly higher than the nonpilot cities (15.19%, *p* = .018). Our findings show that, when implementing information campaigns in nonpilot LTCI cities, policymakers must account for the lower awareness and LTCI policy support in nonpilot cities. This suggests that policymakers should conduct policy advocacy by segmenting the target population, focusing on the less educated and LTCI nonpiloted municipalities. For example, we recommend addressing the lack of policy awareness through the implementation of long-term education campaigns tailored to local conditions and in partnerships with local community-based organizations, to maximize the conversion rate to LTCI among older residents.

There are several limitations. First, we used a convenience sampling method in the third stage of stratified sampling to facilitate sample collection. This sampling method may introduce selection bias, leading to research results that may reflect the characteristics of the accessible population, rather than the whole population, affecting our ability to generalize our results. Although the restrictions we placed on personal characteristics, such as age and sex, enhanced the representativeness of the sample, we recommend that future research consider adopting alternative sampling techniques, like probability sampling methods, to ensure a more comprehensive and unbiased representation of the population. Second, the experimental material for the dementia scenario included the physical incapacitation that may occur in people with Alzheimer’s disease, which overlapped with the disability scenario. Although we have underlined the important information about disability and dementia scenarios in the questionnaires, it reminds future studies to consider other forms of manipulative materials, such as posters and videos, when conducting research on the effects of information interventions, to improve the comparability and generalizability of the findings. Third, the questionnaire only has options related to the subjective intentions to measure the respondents’ intentions to insure LTCI, but the degree of risk perception was not directly measured. Although socioeconomic-demographic characteristics that may affect risk perception were controlled, future studies should include variables to measure the degree of risk overestimation or underestimation. Fourth, our information intervention was completed through an offline face-to-face survey and only provided information about the risks of LTC through the scenarios. Recent studies have noted the effectiveness of information interventions, such as mass media and the Internet, in promoting health risk cognition (46,47). Future research should explore the effectiveness of different types of information intervention measures and the content of information interventions in influencing people’s participation in health-related behaviors. Finally, our study measured respondents’ stated preferences, which may not perfectly reflect their actual health behavior in a complex real world. Although relevant studies have demonstrated a positive correlation between people’s intentions to participate in insurance and their actual behavior (48), the results of this study should be interpreted with caution when generalizing.

## Conclusions

Disability and dementia risk scenario interventions significantly influenced respondents’ LTCI intentions. After information interventions, the disability group’s LTCI intention increased from 43.07% to 79.35%, and the dementia group from 35.54% to 85.54%. The control group only increased their intentions to insure from 42.37% in the pretest to 50.56% in the post-test. The results of heterogeneity of education levels and LTCI pilot/nonpilot policy cities showed that respondents with lower education levels had increased uncertainty about whether to enroll in LTCI under the intervention of disability information, while respondents living in LTCI pilot policy cities had decreased probability of refusing to enroll in LTCI under the intervention of dementia. Government LTCI information campaigns can change people’s risk assessments, increasing the acceptance and uptake of LTCI. We recommend that government LTCI information campaigns differentiate information by dementia-related risks and disability-related risks; by LTCI pilot and nonpilot cities, and by education level.

## Supplementary Material

igaf054_suppl_Supplementary_Materials

## Data Availability

The datasets, analytic methods, and analytic codes used and/or analyzed during the current study are available from the corresponding author on reasonable request. The study was not preregistered.
