# Supplementary material for: Shaping Long-term Care Insurance Intentions Among Chinese Adults Aged 50–70: Role of Information Interventions in Health Risks
Source: Innov Aging. 2025 May 24;9(6):igaf054. doi: 10.1093/geroni/igaf054 (PMC12365492; doi:10.1093/geroni/igaf054)
Supplement: igaf054_suppl_Supplementary_Materials [file igaf054_suppl_supplementary_materials.docx]

***Innovation in Aging* Supplementary Material: Liu, Hao, Maitland, Nicholas, Wang, & Leng. Shaping Long-Term Care Insurance Intentions Among Chinese Adults Aged 50-70: Role of Information Interventions in Health Risks.**

Supplementary Table 1. Measurement of physical health status: Activities of daily living (ADL/IADL) scale

| Items | Completely able to do | Somewhat difficult to do | Need help to do | Completely cannot do |
| --- | --- | --- | --- | --- |
| Take public vehicles | 4 | 3 | 2 | 1 |
| Do housework | 4 | 3 | 2 | 1 |
| Feed yourself | 4 | 3 | 2 | 1 |
| Wash your clothes | 4 | 3 | 2 | 1 |
| Do the shopping | 4 | 3 | 2 | 1 |
| Talk on the phone | 4 | 3 | 2 | 1 |
| Brush your hair or teeth | 4 | 3 | 2 | 1 |
| Prepare a meal | 4 | 3 | 2 | 1 |
| Take medicine | 4 | 3 | 2 | 1 |
| Dress yourself | 4 | 3 | 2 | 1 |
| Wash your body | 4 | 3 | 2 | 1 |
| Get on and off the toilet | 4 | 3 | 2 | 1 |
| Handle own money | 4 | 3 | 2 | 1 |

Supplementary Table 2. Measurement of mental health status: ICECAP-O scale

| Dimension | Content | Degree 1  (for 4 scores) | Degree 2  (for 3 scores) | | Degree 3  (for 2 scores) | | Degree 4  (for 1 scores) |
| --- | --- | --- | --- | --- | --- | --- | --- |
| Attachment | Love and friendship | I can have all the love and friendship I want. | I can have as much love and friendship as I want. | I can only have a little of the love and friendship that I want. | | I can’t have the love and friendship I want. | |
| Security | Thinking about the future without concern | I'll be thinking about the future with no worries. | I'll think about the future with a few concerns. | I'll think about the future with only a few concerns. | | I’ll think about the future with a lot of concerns. | |
| Role | Doing things that make you feel valued | I am able to do all the things that make me feel valuable. | I can do many things that make me feel valuable. | I can do a little of what makes me feel valuable. | | I can’t do anything that makes me feel valuable. | |
| Enjoyment | Enjoyment and pleasure | I can have all the enjoyment and pleasure I want. | I can have as many pleasures as I want. | I can have a few of the pleasures I want. | | I can’t have any of the pleasures I want. | |
| Control | Independence | I can be completely independent. | I can be independent of many things. | I can be independent of some things. | | I’m not independent at all. | |

Supplementary Table 3. Heterogeneity analysis results of educational level

| Variables | Educational level=1 | | | Educational level=2 | | | Educational level=3 | | |
| --- | --- | --- | --- | --- | --- | --- | --- | --- | --- |
|  | (1) Uncertain vs. (2) Yes | (0) No vs. (2) Yes | (0) No vs. (1) Uncertain | (1) Uncertain vs. (2) Yes | (0) No vs. (2) Yes | (0) No vs. (1) Uncertain | (1) Uncertain vs. (2) Yes | (0) No vs. (2) Yes | (0) No vs. (1) Uncertain |
|  | RRR | RRR | RRR | RRR | RRR | RRR | RRR | RRR | RRR |
| Disability intervention | 5.903*** | 0.144*** | 0.024*** | 2.155 | 0.272** | 0.126*** | 1.908 | 0.310* | 0.162*** |
|  | (1.979 - 17.606) | (0.033 - 0.628) | (0.006 - 0.097) | (0.806 - 5.766) | (0.077 - 0.959) | (0.038 - 0.417) | (0.772 - 4.716) | (0.091 - 1.054) | (0.060 - 0.439) |
| Dementia intervention | 11.506*** | 0.202** | 0.018*** | 18.715*** | 1.051 | 0.056*** | 3.011** | 0.357 | 0.119*** |
|  | (4.171 - 31.743) | (0.045 - 0.902) | (0.004 - 0.069) | (4.804 - 72.903) | (0.207 - 5.341) | (0.015 - 0.208) | (1.243 - 7.292) | (0.101 - 1.261) | (0.041 - 0.346) |
| Control variables |  | Yes |  |  | Yes |  |  | Yes |  |
| Constant | 0.547 | 154.656** | 282.876*** | 0.126*** | 374.427** | 119.959*** | 0.567 | 173.408** | 306.079*** |
|  | (0.012 - 25.514) | (2.554 - 9,363.401) | (9.760 - 8,198.319) | (0.038 - 0.417) | (3.265 - 42,940.907) | (3.924 - 3,666.971) | (0.013 - 23.986) | (2.845 - 10,570.363) | (20.340 - 4,605.992) |
| Pseudo R2 |  | 0.3457 |  |  | 0.3168 |  |  | 0.2000 |  |
| Observations | 300 | | |  | 269 |  |  | 456 |  |

*Note.* RRR = Relative Risk Ratio; Confidence intervals in parentheses; ***significant at 1% level; ** significant at 5% level; * significant at 10% level

Supplementary Table 4. Heterogeneity analysis results of LTCI pilot policy

| Variables | LTCI polite policy=0 | | | LTCI polite policy=1 | | |
| --- | --- | --- | --- | --- | --- | --- |
|  | (1) Uncertain vs. (2) Yes | (0) No vs. (2) Yes | (0) No vs. (1) Uncertain | (1) Uncertain vs. (2) Yes | (0) No vs. (2) Yes | (0) No vs. (1) Uncertain |
|  | RRR | RRR | RRR | RRR | RRR | RRR |
| Disability intervention | 2.989** | 0.064*** | 0.191*** | 3.121*** | 0.090*** | 0.281* |
|  | (1.245 - 7.176) | (0.027 - 0.153) | (0.067 - 0.541) | (1.407 - 6.920) | (0.027 - 0.297) | (0.077 - 1.024) |
| Dementia intervention | 11.546*** | 0.042*** | 0.491 | 3.882*** | 0.044*** | 0.170*** |
|  | (3.977 - 33.521) | (0.016 - 0.112) | (0.137 - 1.761) | (1.865 - 8.081) | (0.014 - 0.136) | (0.051 - 0.565) |
| Control variables |  | Yes |  |  | Yes |  |
| Constant | 1.066 | 79.812*** | 85.050*** | 0.575 | 14.312 | 8.232 |
|  | (0.064 - 17.751) | (10.461 - 608.942) | (4.546 - 1,591.075) | (0.014 - 23.405) | (0.059 - 3,487.422) | (0.024 - 2,799.451) |
| Pseudo R2 |  | 0.2848 |  |  | 0.2334 |  |
| Observations |  | 586 |  |  | 439 |  |

*Note.* LTCI = Long-Term Care Insurance; RRR = Relative Risk Ratio; Confidence intervals in parentheses; ***significant at 1% level; ** significant at 5% level; * significant at 10% level
